# Biomarker microRNAs for prostate cancer metastasis: screened with a network vulnerability analysis model

**DOI:** 10.1186/s12967-018-1506-7

**Published:** 2018-05-21

**Authors:** Yuxin Lin, Feifei Chen, Li Shen, Xiaoyu Tang, Cui Du, Zhandong Sun, Huijie Ding, Jiajia Chen, Bairong Shen

**Affiliations:** 1 Center for Systems Biology, Soochow University, Suzhou, 215006 China; 2 Department of Genetics & Systems Biology Institute, Yale University School of Medicine, West Haven, CT 06516 USA; 3 Department of Statistics and Data Science, Yale University, New Haven, CT 06511 USA; 4 Jiangsu Health Vocational College, Nanjing, 211800 China; 5 Suzhou Industrial Park Institute of Services Outsourcing, Suzhou, 215123 China; 6 School of Chemistry, Biology and Material Engineering, Suzhou University of Science and Technology, Suzhou, 215011 China; 7 Center for Translational Biomedical Informatics, Guizhou University School of Medicine, Guiyang, 550025 China; 8 Institute for Systems Genetics, West China Hospital, Sichuan University, Chengdu, 610041 China

**Keywords:** Bioinformatics model, Network vulnerability analysis, MicroRNA biomarkers, Prostate cancer metastasis

## Abstract

**Background:**

Prostate cancer (PCa) is a fatal malignant tumor among males in the world and the metastasis is a leading cause for PCa death. Biomarkers are therefore urgently needed to detect PCa metastatic signature at the early time. MicroRNAs are small non-coding RNAs with the potential to be biomarkers for disease prediction. In addition, computer-aided biomarker discovery is now becoming an attractive paradigm for precision diagnosis and prognosis of complex diseases.

**Methods:**

In this study, we identified key microRNAs as biomarkers for predicting PCa metastasis based on network vulnerability analysis. We first extracted microRNAs and mRNAs that were differentially expressed between primary PCa and metastatic PCa (MPCa) samples. Then we constructed the MPCa-specific microRNA-mRNA network and screened microRNA biomarkers by a novel bioinformatics model. The model emphasized the characterization of systems stability changes and the network vulnerability with three measurements, i.e. the structurally single-line regulation, the functional importance of microRNA targets and the percentage of transcription factor genes in microRNA unique targets.

**Results:**

With this model, we identified five microRNAs as putative biomarkers for PCa metastasis. Among them, miR-101-3p and miR-145-5p have been previously reported as biomarkers for PCa metastasis and the remaining three, i.e. miR-204-5p, miR-198 and miR-152, were screened as novel biomarkers for PCa metastasis. The results were further confirmed by the assessment of their predictive power and biological function analysis.

**Conclusions:**

Five microRNAs were identified as candidate biomarkers for predicting PCa metastasis based on our network vulnerability analysis model. The prediction performance, literature exploration and functional enrichment analysis convinced our findings. This novel bioinformatics model could be applied to biomarker discovery for other complex diseases.

**Electronic supplementary material:**

The online version of this article (10.1186/s12967-018-1506-7) contains supplementary material, which is available to authorized users.

## Background

Prostate cancer (PCa) is one of the common malignant tumors worldwide. In western countries, it has become the second major cause of cancer death among men [1]. The incidence and mortality of this disease were also increasing in Asia during the last decade [2]. At present, the degree of PCa is stratified as low- or high-risk based on the Gleason score, prostate-specific antigen level and other clinical indices. However, such classification seems to be insufficient to monitor PCa progression, especially at the time of metastasis initiation [3]. The early detection of PCa metastatic signature is crucial for evaluating PCa outcome, therefore screening key molecules as biomarkers for predicting PCa metastases is of clinical significance.

MicroRNAs are a group of endogenous, small non-coding RNAs with approximately 22–24 nucleotides in length [4]. They regulate gene expression through base-paring with target messenger RNAs (mRNAs) at the post-transcriptional level and thereby play important roles in a number of important cellular processes [5]. Extensive efforts have been made to identify reliable microRNAs as biomarkers because microRNAs are remarkably stable and specific to be detected in tissues, blood as well as other bodily fluids [6–8]. The expression level of circulating microRNAs also exhibits characteristic alteration in individuals with different pathological settings [9].

Currently, substantial investigations are devoted to discovering microRNA biomarkers for PCa metastasis evaluation, most of which are experimental. Firstly the differentially expressed (DE) or dysregulated microRNAs from large-scale microRNA expression data were extracted as outliers and then the candidates were further validated by low-throughput experiments, such as real-time PCR, etc. [3, 10, 11]. Although experimental methods are powerful enough to detect the abnormal change of microRNA expression between different condition groups, e.g., primary PCa (PPCa) and metastatic PCa (MPCa) [12], it is not easy to identify the driver or key molecules at the systems level. As we known, the mechanism of PCa metastasis is complex, where the dysregulation of microRNAs tends to be highly heterogeneous due to the genetic and environmental factors [13, 14].

Nowadays, computational approaches based on systems biology and network science are well performed on detecting microRNA or gene signatures for the diagnosis and treatment of complex diseases such as cancers [15, 16], diabetes [17] and neurodevelopmental disorders [18]. In particular, Zhang et al. introduced a correlation and clustering based framework to identify microRNA-mRNA network modules for differentiating PPCa and MPCa subtypes [19]. We here aim to develop a novel bioinformatics method to screen single microRNA biomarkers for predicting PCa metastasis, which is propitious to PCa prognosis and therapy.

**Fig. 1:**
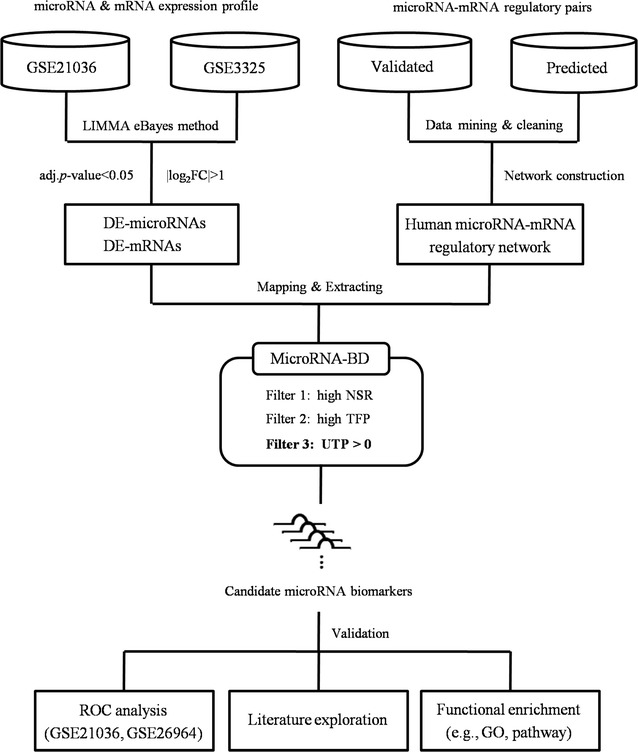
The schematic pipeline for MPCa microRNA biomarker identification. *LIMMA* linear models for microarray data analysis; *eBayes* the empirical bayes; *adj.p value* adjusted *p* value; *FC* fold change; *DE* differentially expressed; *MicroRNA-BD* microRNA biomarker discovery; *NSR* number of single-line regulation; *TFP* transcription factor gene percentage; *UTP* percentage of transcription factor genes in microRNA unique targets; *ROC* receiver operating characteristic curve; *GO* Gene Ontology; *MPCa* metastatic prostate cancer

In our previous studies, we proposed a network-based bioinformatics model called Pipeline of Outlier MicroRNA Analysis (POMA) to detect microRNA biomarkers for cancer diagnosis and prognosis [20–22]. The model integrates microRNA/mRNA expression data with the structural information of microRNA-mRNA regulatory network. Two measurements, i.e., number of single-line regulation (NSR, the number of genes that uniquely regulated by a single microRNA) and transcription factor (TF) gene percentage (TFP, the percentage of TF genes targeted by a single microRNA), were defined to quantify the regulatory power of independent microRNAs. These features characterize network systems vulnerability because the abnormal change of unique regulatory relationships cannot be compensated by other interactions. Statistical evidences demonstrated that microRNAs with significantly high NSR and TFP values are more likely to be biomarkers [20, 21]. In this study, we improved and updated the model as MicroRNA Biomarker Discovery (MicroRNA-BD) by analyzing the network vulnerability and considering the functional importance of genes that are uniquely regulated by given microRNAs. We then applied the model to screen key microRNAs as biomarkers for predicting PCa metastasis. The schematic pipeline of the MicroRNA-BD model is shown in Fig. 1.

## Methods

### Data collection

The microRNA and mRNA expression dataset for biomarker prediction (GSE21036 [23] and GSE3325 [24]) were downloaded from gene expression omnibus (GEO) [25]. Among them, GSE21036 was generated by Agilent-019118 Human microRNA Microarray 2.0 G4470B, and contained microRNA expression data from 142 prostate tissue samples. Here 113 of the samples including 99 PPCa and 14 MPCa were extracted for further analysis. The mRNA expression dataset GSE3325 was performed on Affymetrix Human Genome U133 Plus 2.0 Array, 5 and 4 individual samples for PPCa and MPCa were selected, respectively. Moreover, another independent dataset GSE26964 [26] with 6 PPCa and 7 MPCa samples from Capitalbio mammal microRNA V3.0 platform was selected for result validation. The details are summarized in Table 1.

**Table 1:** Summary of the microRNA and mRNA dataset used in this study

Category	RNA type	GEO accession	Platform	Sample source	Number of samples (PPCa/MPCa)
Prediction	microRNA	GSE21036	GPL8227	Prostate tissue	113 (99/14)
	mRNA	GSE3325	GPL570	Prostate tissue	9 (5/4)
Validation	microRNA	GSE26964	GPL8469	Prostate tissue	13 (6/7)

*PPCa* primary prostate cancer; *MPCa* metastatic prostate cancer

Besides, the previously reported PCa microRNA biomarkers were manually collected and integrated from: (1) the review for PCa microRNAs by Vanacore et al. [27], (2) NCBI PubMed using the retrieval terms as “(prostate cancer[tiab]) AND (microRNA*[tiab] OR miRNA*[tiab]) AND (biomarker*[tiab] OR marker*[tiab] OR indicator*[tiab] OR predict*[tiab])”.

### Differentially expressed microRNAs (DE-microRNAs) and mRNAs (DE-mRNAs) extraction

The DE-microRNAs and DE-mRNAs were extracted based on the comparison of their expressions between PPCa and MPCa samples using the empirical bayes (eBayes) method in linear models for microarray data analysis (LIMMA) R package [28, 29]. The Benjamini–Hochberg false discovery rate method was applied to adjust raw *p* values [30]. For the gene that is related to multiple probes, we assigned it to the probe that had the most significant variation across its expression profile. The adjusted *p* value (adj.*p* value) < 0.05 and |log_2_fold-change| > 1 were chosen as the cut-off.

### MPCa-specific microRNA-mRNA network construction

The MPCa-specific microRNA-mRNA network was constructed in two steps: First, a human microRNA-mRNA network (termed as the reference network) was built based on both experimentally validated and computationally predicted microRNA-mRNA regulatory data. Compared with our previous work [20, 21], the microRNA-mRNA pairs were updated and moreover, the latest nomenclature of microRNAs in miRBase (Release 21) [31] was fully considered before network reconstruction. Here the experimental data were mined from miRTarBase (version 4.5) [32], TarBase (version 6.0) [33], miRecords (version 4.0) [34], and miR2Disease [35] whereas the predicted data included information from HOCTAR (version 2.0) [36], ExprTargetDB [37], and starBase (version 2.0) [38]. To reduce the false positive rate, microRNA-mRNA pairs validated by low-throughput experiments, e.g, real-time PCR etc. were considered in this study while the predicted pairs were selected only when they existed at least in two of the three computational prediction databases. In the second step, the DE-microRNAs and DE-mRNAs were mapped onto the reference to extract the MPCa-specific microRNA-mRNA network.

### Biomarker microRNA identification based on network vulnerability analysis

**Fig. 2:**
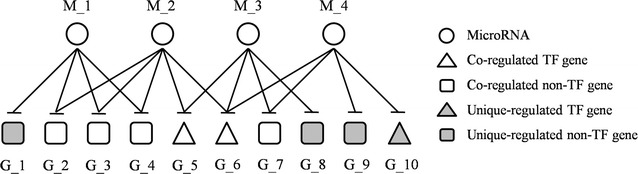
Schematic description of microRNA-mRNA regulatory types. Four types were defined here, i.e., TF or non-TF genes regulated by multiple or single microRNAs. For example, *G_1* was uniquely regulated by *M_1* whereas TF gene *G_5* was co-regulated by *M_2* and *M_3*. The co-regulatory sites are robust since one of the regulations altered can be compensated by others. Here the unique regulatory sites, i.e., single-line regulations, are considered as the vulnerable structure in the network. Meanwhile, microRNAs that target more TF genes seem to be functionally important. *M* microRNA; *G* gene; *TF* transcription factor

As illustrated in Fig. 2, the microRNA-mRNA relationship can be classified into four types based on their regulatory modes. The POMA model proposed in our previous studies pays more attention to the single-line regulatory power of microRNAs and focuses on the biological functions of their targets [20, 21]. After analyzing the sub-structure of microRNA-mRNA network, we found that microRNAs still held the potential to uniquely regulate genes with crucial functions, e.g., transcription factor (TF) genes. As an example, the TF gene *G_10* in Fig. 2 is uniquely regulated by *M_4*. The alteration of single-line regulation is not compensated, and TFs are often key players in many important biological processes, thus it is reasonable to assume that the dysregulation of such regulatory patterns is more likely to alter the system stability and eventually cause the systematic disorder. To strengthen the importance of microRNA regulation on TF genes, we here applied a novel parameter called the unique-regulated TF gene percentage (UTP). Numerically, it is equivalent to the percentage of TF genes in the microRNA unique targets. Finally, our previous model POMA was improved as MicroRNA-BD to identify microRNA biomarkers with the following three measurements for network vulnerability characterization.

Step 1: NSR (number of single-line regulation) measurement is the number of genes that are uniquely regulated by a single microRNA. MicroRNAs with significantly high NSR values (p value < 0.05, Wilcoxon signed-rank test) were extracted based on the analysis of MPCa-specific microRNA-mRNA network
Step 2: TFP (transcription factor gene percentage) measurement is the percentage of TF genes targeted by a single microRNA. MicroRNAs with significantly high TFP values (p value < 0.05, Wilcoxon signed-rank test) were selected from those screened in *Step 1*;
Step 3: UTP (unique-regulated TF gene percentage) measurement is defined as the percentage of TF genes in microRNA unique targets. MicroRNAs with UTP > 0 in *Step 2* were identified as candidate biomarkers.

### Performance evaluation

We performed the receiver-operating characteristic (ROC) analysis on both prediction and validation microRNA datasets to evaluate the performance of identified microRNA biomarkers for classifying MPCa and PPCa. The ROC curve and the area under curve (AUC) were drawn and calculated for each of the identified microRNAs using the R package ‘epicalc’ [39]. The percentage of the reported MPCa microRNA biomarkers in the predicted set was defined as prediction precision to quantify the performance of the model.

### Functional enrichment analyses

To validate the association between the targets of candidate microRNA biomarkers and PCa metastasis, we performed Gene Ontology (GO) annotation, Kyoto Encyclopedia of Genes and Genomes (KEGG) [40] pathway analyses and Ingenuity Pathway Analysis (IPA) [41] using Database for Annotation, Visualization and Integrated Discovery (DAVID, version 6.7) [42] and IPA program [41], respectively. Here the targets of identified microRNA biomarkers were retrieved from human microRNA-mRNA network and Benjamini–Hochberg method was used to adjust raw *p* values for enrichment analysis. The top ten significantly enriched terms (adj.*p* value < 0.05) were selected and further studied for their correlations with PCa metastasis through literature validation.

## Results

### Biomarker microRNAs for predicting PCa metastasis

**Fig. 3:**
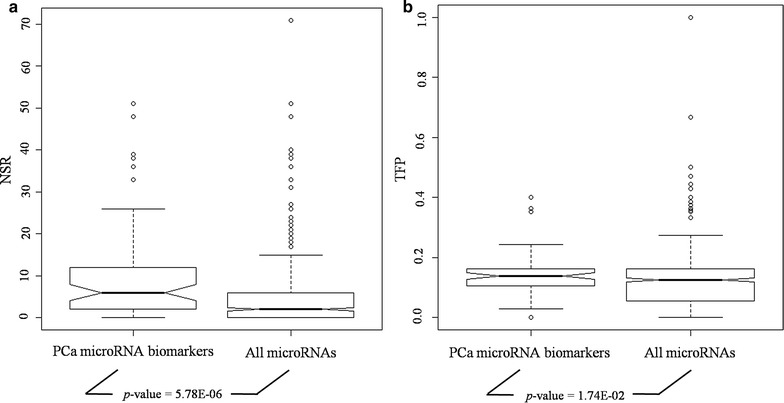
Topological and functional characterization of reported PCa microRNA biomarkers. **a** NSR distribution of reported PCa microRNA biomarkers and all microRNAs in human microRNA-mRNA network. **b** TFP distribution of reported PCa microRNA biomarkers and all microRNAs in human microRNA-mRNA network. The statistical significance was calculated using Kolmogorov–Smirnov test. *NSR* number of single-line regulation; *TFP* transcription factor gene percentage; *PCa* prostate cancer

A total of 67 literature reported PCa microRNA biomarkers were manually collected (see Additional file 1). The human microRNA-mRNA network included 48,868 regulatory pairs among 618 microRNAs and 9526 genes/mRNAs. As shown in Fig. 3, Additional files 1 and 2, respectively, PCa biomarker microRNAs had significant regulatory power in the network, which convinced the priori evidence for biomarker discovery [20, 21]. Moreover, more than 53.7% (36/67) of these microRNAs had UTP > 0, which was approximately twice greater than that in the whole network (27.5%, 170/618).

Based on the selected sample data, 91 DE-microRNAs and 970 DE-mRNAs were statistically identified. The MPCa-specific microRNA-mRNA network contained 505 microRNA-mRNA regulatory pairs among 65 microRNAs and 263 mRNAs (see Additional file 3). Seven microRNAs, i.e., miR-204-5p, miR-101-3p, miR-145-5p, miR-198, miR-152, miR-130a-3p and miR-363-3p tended to have significantly high NSR and TFP values in MPCa-specific microRNA-mRNA network (see Additional file 4). The UTP measurements for miR-130a-3p and miR-363-3p are 0, therefore, the remaining five microRNAs, i.e., miR-204-5p, miR-101-3p, miR-145-5p, miR-198, and miR-152 were screened as putative biomarkers for predicting PCa metastasis after MicroRNA-BD filtration.

**Fig. 4:**
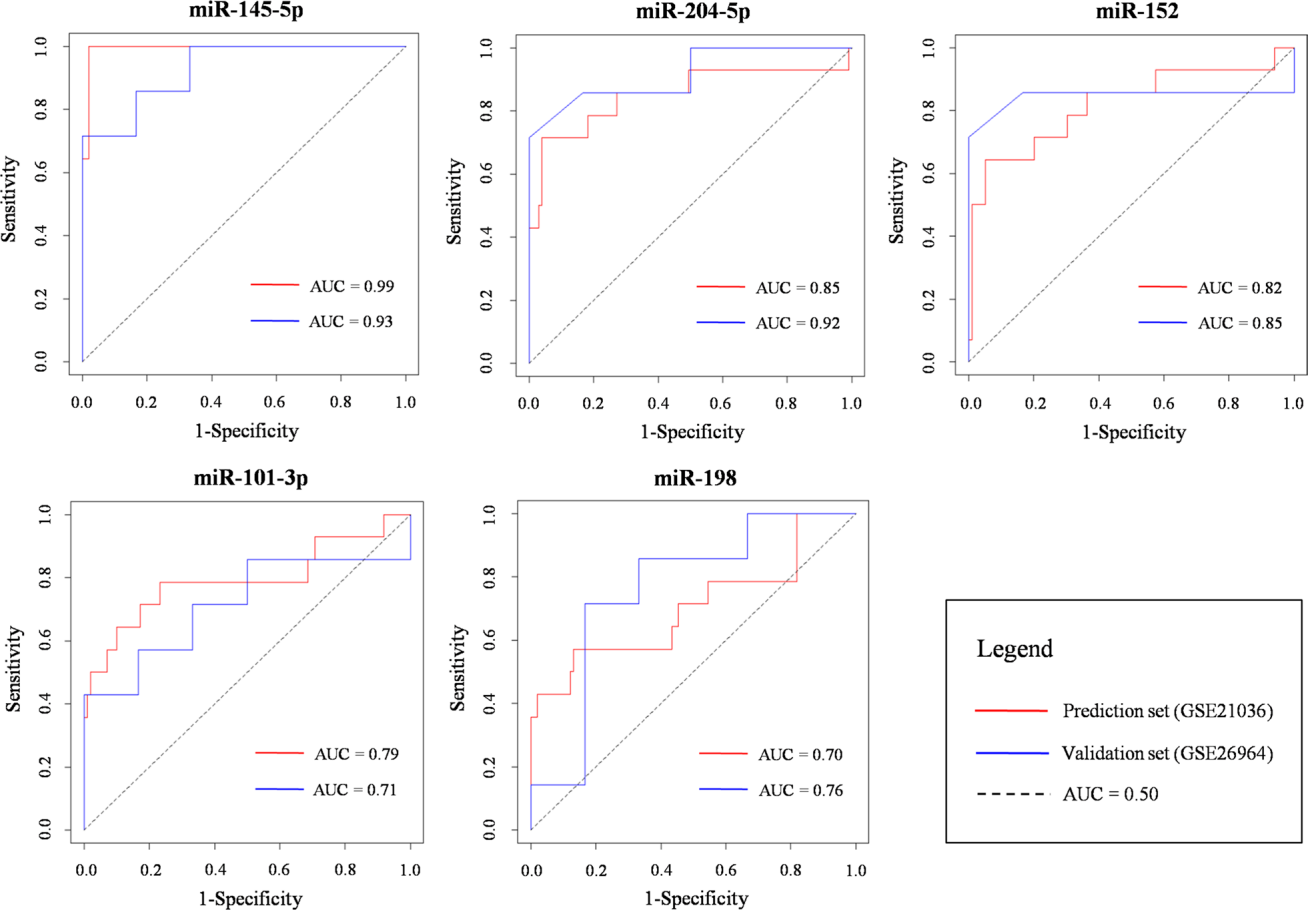
ROC analysis for the identified microRNA biomarkers. The AUC distribution in the prediction set GSE21036 and another independent validation set GSE26964 ranged from 0.70 to 0.99 and from 0.71 to 0.93, respectively. Red curve: GSE21036; blue curve: GSE26964. *PPCa* primary prostate cancer; *MPCa* metastatic prostate cancer; *ROC* receiver operating characteristic curve; *AUC* area under the curve

As shown in Table 2, four of the identified microRNAs, i.e., miR-204-5p, miR-101-3p, miR-145-5p, and miR-152, were significantly down-regulated in MPCa group whereas miR-198, was over-expressed in MPCa samples compared with PPCa. The ROC curves for their PCa metastasis prediction performance were shown in Fig. 4. In the prediction set GSE21036 and another independent validation set GSE26964, the AUC ranged from 0.70 to 0.99 and from 0.71 to 0.93, respectively. Overall, miR-145-5p, miR-204-5p, and miR-152 achieved the best performance (AUC > 0.80) on PPCa and MPCa subtyping, and the AUC distribution of the five microRNAs in two datasets was highly consistent, which indicated the predictive power of the identified microRNA biomarkers for discriminating between MPCa and PPCa.

**Table 2:** Details for the identified microRNA biomarkers

microRNA ID	Adj.*p* value (PPCa vs MPCa)	log_2_ (FC)	Target number	NSR	TFP	UTP
miR-204-5p	3.43E−08	− 2.0896	21	13	0.2857	0.3846
miR-101-3p	1.26E−08	− 1.0684	24	3	0.2917	0.3333
miR-145-5p	8.00E−25	− 3.2157	12	3	0.2500	0.3333
miR-198	7.67E−05	1.2564	12	5	0.3333	0.2000
miR-152	3.67E−08	− 1.0146	17	6	0.2941	0.1667

*PPCa* primary prostate cancer; *MPCa* metastatic prostate cancer; *adj. p value* adjusted *p* value; *FC* fold change; *NSR* number of single-line regulation; *TFP* transcription factor gene percentage; *UTP* percentage of transcription factor genes in microRNA unique targets

### Literature validation of the identified microRNA biomarkers

All of the five microRNAs were involved in PCa pathogenesis according to PubMed literature reports. In particular, two microRNAs (40%, 2/5), i.e., miR-145-5p and miR-101-3p, have been reported as potential biomarkers for human PCa metastasis [27, 43, 44]. As a tumor suppressor microRNA, the down-regulation of miR-145 (namely miR-145-5p) could cause cell invasion and migration in PCa progression [27]. Huang et al. showed that miR-145 regulated the characteristics of cancer stem cells and played important roles in the progression of PCa bone metastasis [45]. Moreover, miR-145 is a direct target of p53, and the loss of wild-type p53 could promote PCa bone metastasis by partially repressing miR-145 expression [46]. Chakravarthi et al. showed that the loss of miR-101 (namely miR-101-3p) may affect the expression of SUB1 and lead to the activation of known oncogenes driving PCa metastasis [47]. Besides, miR-204-5p and miR-152 were also associated with PCa progression and metastasis. Lin et al. [48] found that miR-204-5p was a tumor suppressor and could promote apoptosis through regulating BCL2 in PCa cells. Todorova et al. studied the effect of miR-204 (namely miR-204-5p) modulation on important TFs for PCa bone marrow metastasis and uncovered that this microRNA was dysregulated in MPCa in vitro [49]. Theodore et al. analyzed the microRNA expression profile data and found the ethnic difference of miR-152 expression between African American (AA) and Caucasian PCa patients. On the other hand, the epigenetic regulation of miR-152 and DNMT1 may contribute to the aggressiveness of PCa tumors, especially to AA PCa patients [50]. Last but not least, miR-198 was found to be up-regulated in high grade (Gleason score ≥ 8) PCa tumors, which would help recognize the aggressive behavior of PCa [51]. However, the filtered two microRNAs, i.e., miR-130a-3p and miR-363-3p, did not show any reliable correlation with PCa metastasis so far. In summary, MicroRNA-BD outperformed previous models and improved the microRNA biomarker prediction precision from 28.6% (2/7) to 40% (2/5) in this case study.

**Fig. 5:**
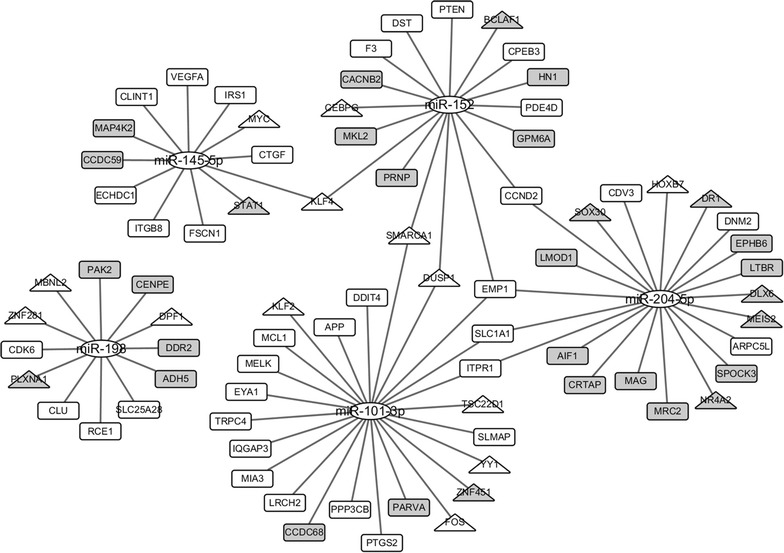
Identified biomarker microRNAs and their targets in MPCa-specific microRNA-mRNA network. Elliptic, triangular and rectangular nodes represent microRNAs, TF genes and non-TF genes, respectively. Nodes in grey represent genes that are uniquely regulated by single microRNAs in the network. *MPCa* metastatic prostate cancer; *TF* transcription factor

We further evaluated the relationship between the targets of identified microRNA biomarkers and PCa metastasis. As illustrated in Fig. 5, the TF gene STAT1, which was uniquely regulated by miR-145-5p in the MPCa-specific microRNA-mRNA network, could be activated by Endoglin. In the study, Endoglin was shown to suppress the cell invasion of PCa and inhibit PCa metastasis [52]. Meanwhile, several uniquely-regulated non-TF genes, such as DDR2 and MRC2, also participated in the PCa metastatic progression [53, 54]. For instance, DDR2 regulated the promoter activity of parathyroid hormone-related protein and thereby facilitated the bone metastasis of PCa [53]. In addition, EMP1 suppressed PCa cell proliferation and invasion by regulating caspase-9 and VEGFC protein [55], and it was co-regulated by miR-101-3p, miR-204-5p and miR-152 in the network. Some other co-regulated genes, including PTEN and MYC, were also functional during PCa evolution [56, 57].

### Gene ontology (GO) annotation

The GO analysis was performed using the online tool DAVID at three levels, i.e., biological process (BP), cellular component (CC), and molecular function (MF). The statistically significant terms (adj.*p* value < 0.05) of each level were summarized in Additional file 5. Here we mainly focused on the top ten significantly enriched terms for in-depth analyses. As shown in Table 3, at the BP level, the most significant terms were closely relevant to cell cycle, metabolic processes and cell death. Accumulating evidence demonstrated that a number of genes as well as non-coding RNAs played functions in PCa metastasis by dramatically activating or inhibiting the cell cycle process [58–60]. Wang et al. analyzed the gene expression data of castration-resistant PCa and found that the identified regulatory modules were also enriched in the phosphorus metabolic process [61]. At the CC level, the enriched terms were mainly concentrated on nuclear lumen, nucleoplasm, and organelle lumen. Nucleophosmin (NPM1) is a nucleoprotein and associated with tumor growth. Destouches et al. showed that the phosphorylated NPM1 may interact with androgen receptor in nucleoplasm, which is biologically important in PCa progression [62]. At the MF level, most enriched terms were linked with molecular activities, including transcription regulator activity, transcription repressor activity, transcription activator activity, etc. Grubb RL et al. found that the transcription regulatory protein STAT3 differed statistically in PCa with high Gleason grade (≥ 8) [63]. Xiao et al. showed that the loss expression of PLZF, a transcription repressor in oncogenesis, correlated with PCa tumor aggressiveness [64], which highlighted the functional importance of transcription repressor activity in PCa metastasis.

**Table 3:** Top ten significant GO terms enriched by targets of the identified microRNA biomarkers

Category	GO terms	Number of enriched genes	Adj.*p* value
BP	Mitotic cell cycle	60	3.52E−07
	Phosphorus metabolic process	112	4.00E−06
	Phosphate metabolic process	112	4.00E−06
	Regulation of apoptosis	97	3.51E−06
	Positive regulation of cell proliferation	61	2.83E−06
	Cell cycle process	75	3.16E−06
	Regulation of programmed cell death	97	2.92E−06
	Regulation of cell death	97	3.02E−06
	Cell cycle	93	4.11E−06
	Regulation of transcription from RNA polymerase II promoter	88	6.10E−06
CC	Nuclear lumen	162	1.17E−13
	Nucleoplasm	110	7.25E−12
	Organelle lumen	182	1.05E−11
	Membrane-enclosed lumen	183	2.45E−11
	Intracellular organelle lumen	176	5.37E−11
	Nucleoplasm part	77	1.95E−10
	Intracellular non-membrane-bounded organelle	212	5.33E−06
	Non-membrane-bounded organelle	212	5.33E−06
	Chromatin remodeling complex	18	3.26E−05
	Nucleolus	72	1.81E−04
MF	Transcription regulator activity	163	1.16E−09
	Transcription repressor activity	50	1.35E−06
	Transcription factor binding	67	4.45E−06
	Transcription activator activity	56	1.36E−05
	Transcription factor activity	100	1.41E−04
	Protein kinase activity	68	5.01E−04
	Transcription cofactor activity	46	1.07E−03
	Protein serine/threonine kinase activity	50	3.67E−03
	DNA binding	190	5.00E−03
	Phosphoprotein phosphatase activity	25	8.40E−03

*GO* gene ontology; *BP* biological process; *CC* cellular component; *MF* molecular function; *adj.p value*: adjusted *p* value

### Pathway enrichment analysis

**Fig. 6:**
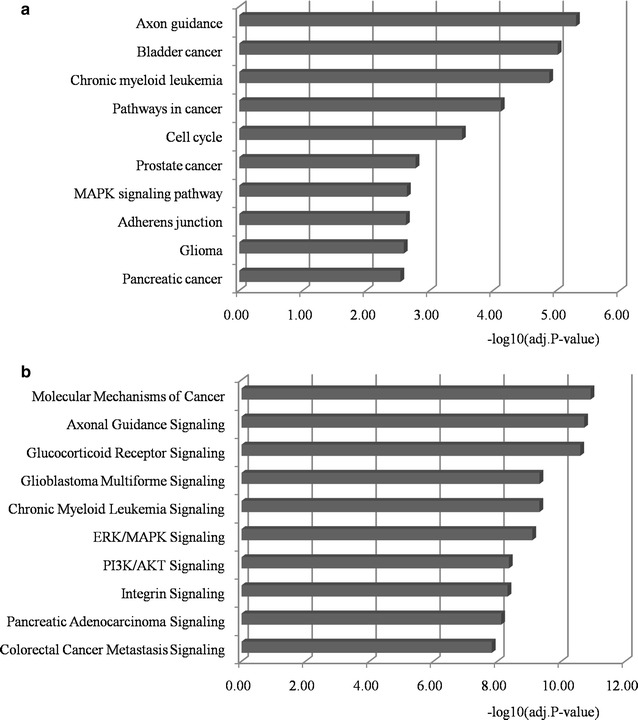
Pathway enrichment analysis for targets of the identified microRNA biomarkers. The statistical significance level (adj. *p* value) was negative 10-based log transformed. **a** The top ten significant KEGG terms. **b** The top ten significant IPA terms. *adj.p value* adjusted *p* value; *KEGG* Kyoto Encyclopedia of Genes and Genomes; *IPA* ingenuity pathway analysis

**Fig. 7:**
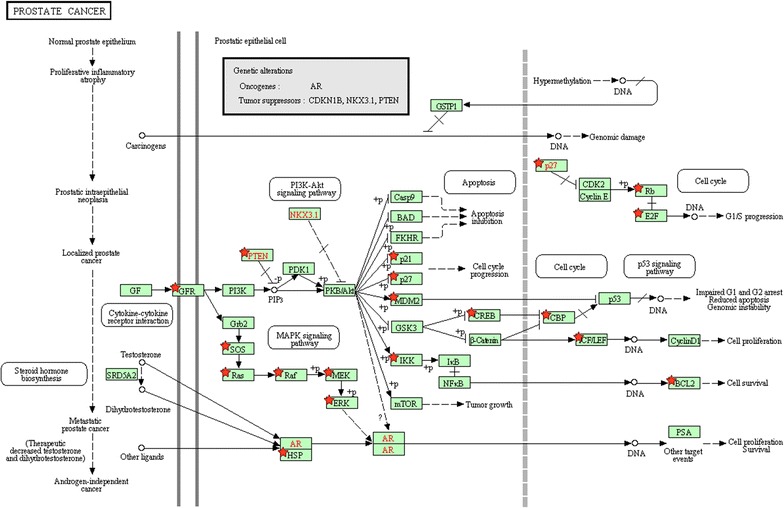
The prostate cancer pathway enriched in KEGG. Objects with pentagrams are acting locus by mapped genes. *KEGG* Kyoto Encyclopedia of Genes and Genomes

To investigate the functional mechanisms of the five microRNA candidates, we performed the KEGG and IPA pathway enrichment analysis on their targets using DAVID and IPA program, respectively. The significantly enriched terms (adj.*p* value < 0.05) were listed in Additional files 6 and 7, respectively. Here the top ten significant terms were mainly selected for further literature exploration. As shown in Fig. 6a, the most meaningful KEGG terms were Axon guidance, Pathways in cancer, Cell cycle, Prostate cancer, and MAPK signaling pathway. Ding et al. found that Semaphorin 4F (S4F), which played important roles in embryologic axon guidance, was a key regulator in the tumor microenvironment and a biomarker of aggressive PCa [65]. McNair et al. uncovered that the cell cycle-coupled expansion of AR activity promoted the progression of PCa and was related to the development of PCa metastases [66]. As shown in Fig. 7, the targets enriched in the prostate cancer pathway had close relations with cell cycle. They potentially mediated the process of cell proliferation and cell survival. Two tumor suppressors, i.e., PTEN and p27 (CDKN1B), which showed the prognostic or therapeutic value in PCa metastasis and recurrence [56, 67], were functionally regulated by the identified microRNAs.

**Fig. 8:**
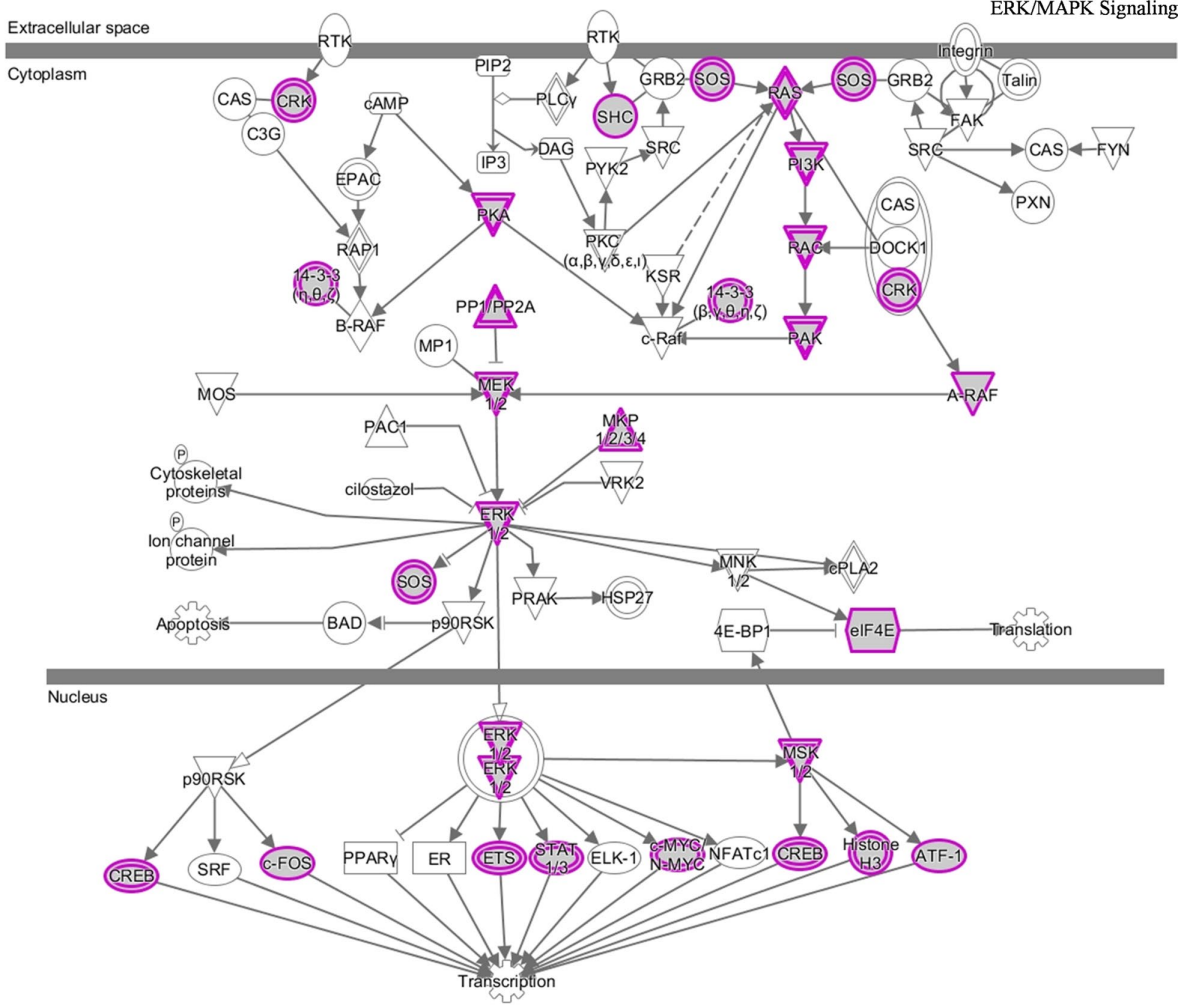
The ERK/MAPK signaling enriched in IPA. Objects with purple circles or triangles are acting locus by mapped genes. *ERK* extracellular signal-regulated kinases; *MAPK* mitogen-activated protein kinase; *IPA* ingenuity pathway analysis

As shown in Fig. 6b, several pathways were also significantly enriched in IPA, such as molecular mechanisms of cancer, axonal guidance signaling, and ERK/MAPK signaling etc. Substantial efforts have convinced that the MAPK signaling was involved in the progression of advanced or metastatic PCa [68–70]. As illustrated in Fig. 8, the targets of identified biomarkers were almost enriched in the hubs of ERK/MAPK signaling, which demonstrated the regulatory power of these microRNAs. Chen et al. reviewed the relation between PI3K/AKT signaling and PCa tumorigenesis and pointed out that this pathway regulated tumor cell invasion during the metastasis of PCa cells [71]. Kassi et al. reported that glucocorticoids could mediate the transcriptional regulation of genes which were functional in PCa cell growth, inflammation, differentiation, apoptosis, and metastasis, and the glucocorticoids receptor signaling participated in PCa through cross talking with other signaling cascades [72]. Another well-studied pathway associated with PCa development is TGF-β signaling. It is reported that the TGF-β pathway held the potential to maintain tissue homeostasis and was functional during cancer cell proliferation [73]. Bonci et al. found that the concomitant decrease of miR-15/16 and increase of miR-21 could abnormally activate TGF-β signaling, leading to the invasion, migration and distant bone metastasis of PCa cells [12]. Zhang et al. demonstrated that the TGF-β pathway was significantly enriched by genes in the identified biomarker modules for PCa subtyping [19]. In this study, as illustrated in Additional files 7 and 8, respectively, the TGF-β signaling was also shown statistical significance as the target of the five microRNAs. Moreover, TGFBR1 and TGFBR2, two transforming growth factor beta receptors with the power of transferring TGF-beta signal from the extracellular space to the cytoplasm, were closely regulated by these microRNAs, which could strengthen the importance of our findings.

We noticed that the targets of the identified microRNAs were also correlated with bladder cancer, glioma, pancreatic cancer and colorectal cancer, which indicated the similarity of pathogenesis between PCa metastasis and other cancers. For example, TRAP1/Hsp75 has been reported to be a molecular marker in metastatic PCa. Li et al. proved that the expression of TRAP1 was increased in glioma compared with its normal controls, and it could be a useful prognostic factor in glioma management [74]. The RNASEL germline variants were associated with not only familial PCa, but also pancreatic cancer, which indicated the potential mechanisms between pancreatic cancer and PCa development [75].

## Discussion

PCa is a commonly diagnosed cancer among males around the world. While the overall survival rate has increased these years, the metastasis is still a leading cause of PCa death [76]. The early detection of metastatic signature is important for monitoring PCa prognosis and helping design personalized therapeutic strategies. It is widely acknowledged that microRNAs are a class of functional regulators in biological processes and have good sensitivity and specificity to be biomarkers for disease initiation and progression [77].

In this study, we integrated microRNA/mRNA expression data with network structural knowledge and improved the bioinformatics model to screen candidate microRNA biomarkers for predicting PCa metastasis. Compared with the previous work, we updated the reference microRNA-mRNA network by carefully integrating recently reported human microRNA-mRNA associations based on microRNA nomenclature. Besides the single-line regulatory power (NSR) and biological roles of microRNAs (TFP), we considered the functional importance of genes that are uniquely regulated by single microRNAs in this model. It is reasonable that the single-line regulatory site in the network is relatively vulnerable and TFs are important regulators in various biological activities. The incorporative analysis of structural and functional characteristics in microRNA-mRNA regulation would strengthen the evalutaion of microRNA regulatory power. Hence we defined a new parameter called UTP to quantify the special regulation of given microRNAs, that is, the percentage of TF genes in microRNA unique targets.

Based on this computational model, a total of five microRNAs, i.e., miR-204-5p, miR-101-3p, miR-145-5p, miR-198, and miR-152, were identified as candidate biomarkers for PCa metastasis prediction. Among them, miR-145-5p and miR-204-5p were validated as tumor suppressor microRNAs, and their down-regulation could disorder cell cycle processes and finally result in PCa invasion [27, 48]. More importantly, miR-145-5p and miR-101-3p have been reported as biomarkers for evaluating PCa metastasis previously [43, 44]. The remaining three were also confirmed to be connected with the development of metastatic or high-grade PCa according to PubMed literature searches. Compared with POMA solely using NSR and TFP as filters, the MicroRNA-BD model improved the prediction precision from 28.6% (2/7) to 40% (2/5) in this case study. Furthermore, the AUC values for the prediction performance of the five microRNAs, respectively, ranged from 0.70 to 0.99 and from 0.71 to 0.99 in two datasets, i.e., the prediction set and another independent validation set, which indicated the power of the identified biomarkers for PCa prognosis and metastasis tracking.

We further investigated the pathogenic mechanisms of the identified microRNAs in PCa metastasis through GO and pathway enrichment analyses. The cell cycle process, for example, is one of the most significant terms enriched in GO and KEGG pathway, which supported the pivotal view of cell cycle-mediated PCa carcinogenesis [66, 78, 79]. The prostate cancer pathway, ERK/MAPK signaling, and TGF-β signaling are well-studied in PCa metastasis, and we found that most of the targets of identified microRNA biomarkers were the key components of these pathways, meanwhile, some tumor suppressor genes, e.g., PTEN and p27, were regulated by the identified microRNAs. In addition, the remaining pathways, such as axonal guidance [65], PI3K/AKT signaling [71], glucocorticoids receptor signaling [72], pancreatic cancer [75], and molecular mechanisms of cancer [80] etc., were all involved in PCa progression and metastasis according to previous reports.

Compared with the existing approaches for PCa metastasis investigation, our model detects microRNA biomarkers based on statistical evidences from a combination of network sub-structural and functional analyses. In the study of Zhang et al., five microRNA-mRNA modules were identified for PPCa and MPCa respectively based on the correction and clustering analysis on microRNA and mRNA datasets [19]. Similar to this idea, our study also utilized microRNA-mRNA network information. However, the network in our model was concentrated more as a biological system, where special regulatory patterns altering its stability were concluded as the principle for biomarker prediction. Another major difference between the two approaches regards to the methodology for sub-network extraction. In contrast to the two types of network modules, i.e., PPCa- and MPCa-module, built in Zhang et al. using clustering and condensing techniques, [19], our model constructed microRNA-mRNA network specific to PCa metastasis in order to capture the changing signatures during PCa evolution, and only single microRNAs could be screened as candidate biomarkers for PCa subtyping. Considering the results, the miR-145-5p detected by our model was also involved in one of the modules in Zhang et al. [19], which convinced the underlying power of this microRNA for PCa metastasis predicting. From pathway angles, both Zhang et al. [19] and Bonci et al. [12] demonstrated the importance of TGF-β signaling as microRNA targets in PCa progression and metastasis. As described in the section of ‘Pathway enrichment analysis’, we found that genes regulated by the five biomarker microRNAs were similarly enriched in TGF-β signaling, which indicated the pathway-level consistency of results for PCa carcinogenesis decoding across these studies. Due to the complexity and diversity in PCa development, biomarker microRNAs identified by different methods tended to be highly heterogeneous. In systems biology viewpoints, living organisms are often treated as a holistic framework, thus identifying module or network biomarkers catering to the dynamical nature of PCa pathogenesis for personalized prognosis and treatment is our next-step action.

It should not be ignored that some limitations still existed in this study. Firstly, genes in the present model were treated equally. As we known, the importance of genes in different biological activities is not the same, more functional annotations need to be weighted reasonably. Secondly, only TF genes were selected in the study, the specific knowledge to PCa metastasis could be considered in order to provide precise strategies for MPCa early detection and treatment. Thirdly, only 618 microRNAs were recorded in our reconstructed human microRNA-mRNA network, the network scale should keep pace with the development of newly identified microRNA-mRNA associations. Last but the most important, we are trying to collect human PPCa and MPCa samples to further perform wet lab verifications for future carcinogenic exploration and translational application.

## Conclusion

In this study, a total of five microRNAs, i.e, miR-204-5p, miR-101-3p, miR-145-5p, miR-198, and miR-152, were identified as candidate biomarkers for predicting PCa metastasis based on a novel bioinformatics model. The prediction performance, literature exploration and functional enrichment analysis convinced our findings. More clinical validations are needed in our future translational application.

## Additional files

**Additional file 1.** Literature-reported PCa microRNA biomarkers.

**Additional file 2.** The NSR, TFP and UTP values for microRNAs in the reconstructed human microRNA-mRNA network.

**Additional file 3.** MPCa-specific microRNA-mRNA network.

**Additional file 4.** MicroRNAs with significantly high NSR and TFP values in MPCa-specific microRNA-mRNA network. The *p* values were calculated using Wilcoxon signed-rank test.

**Additional file 5.** Significant GO terms enriched by targets of the identified microRNA biomarkers. The *p* values were adjusted using Benjamini–Hochberg method.

**Additional file 6.** Significant KEGG terms enriched by targets of the identified microRNA biomarkers. The *p* values were adjusted using Benjamini–Hochberg method.

**Additional file 7.** Significant IPA terms enriched by targets of the identified microRNA biomarkers. The *p* values were adjusted using Benjamini–Hochberg method.

**Additional file 8.** The TGF-β signaling enriched in IPA. Objects with purple circles or triangles were acting locus by mapped genes.

## Abbreviations

PCa: prostate cancer
PPCa: primary prostate cancer
MPCa: metastatic prostate cancer
LIMMA: linear models for microarray data analysis
eBayes: the empirical bayes
adj.*p* value: adjusted *p* value
FC: fold change
DE: differentially expressed
TF: transcription factor
MicroRNA-BD: microRNA biomarker discovery
POMA: pipeline of outlier microRNA analysis
NSR: number of single-line regulation
TFP: transcription factor gene percentage
UTP: unique-regulated TF gene percentage
ROC: receiver operating characteristic curve
AUC: area under the curve
GO: gene ontology
BP: biological process
CC: cellular component
MF: molecular function
KEGG: Kyoto Encyclopedia of Genes and Genomes
DAVID: Database for Annotation, Visualization and Integrated Discovery
IPA: ingenuity pathway analysis
