# Supplementary material for: Biomarker microRNAs for prostate cancer metastasis: screened with a network vulnerability analysis model
Source: J Transl Med. 2018 May 21;16:134. doi: 10.1186/s12967-018-1506-7 (PMC5963164; doi:10.1186/s12967-018-1506-7)
Supplement: Supplementary file 8 — Additional file 8. The TGF-β signaling enriched in IPA. Objects with purple circles or triangles were acting locus by mapped genes. [file 12967_2018_1506_MOESM8_ESM.doc]

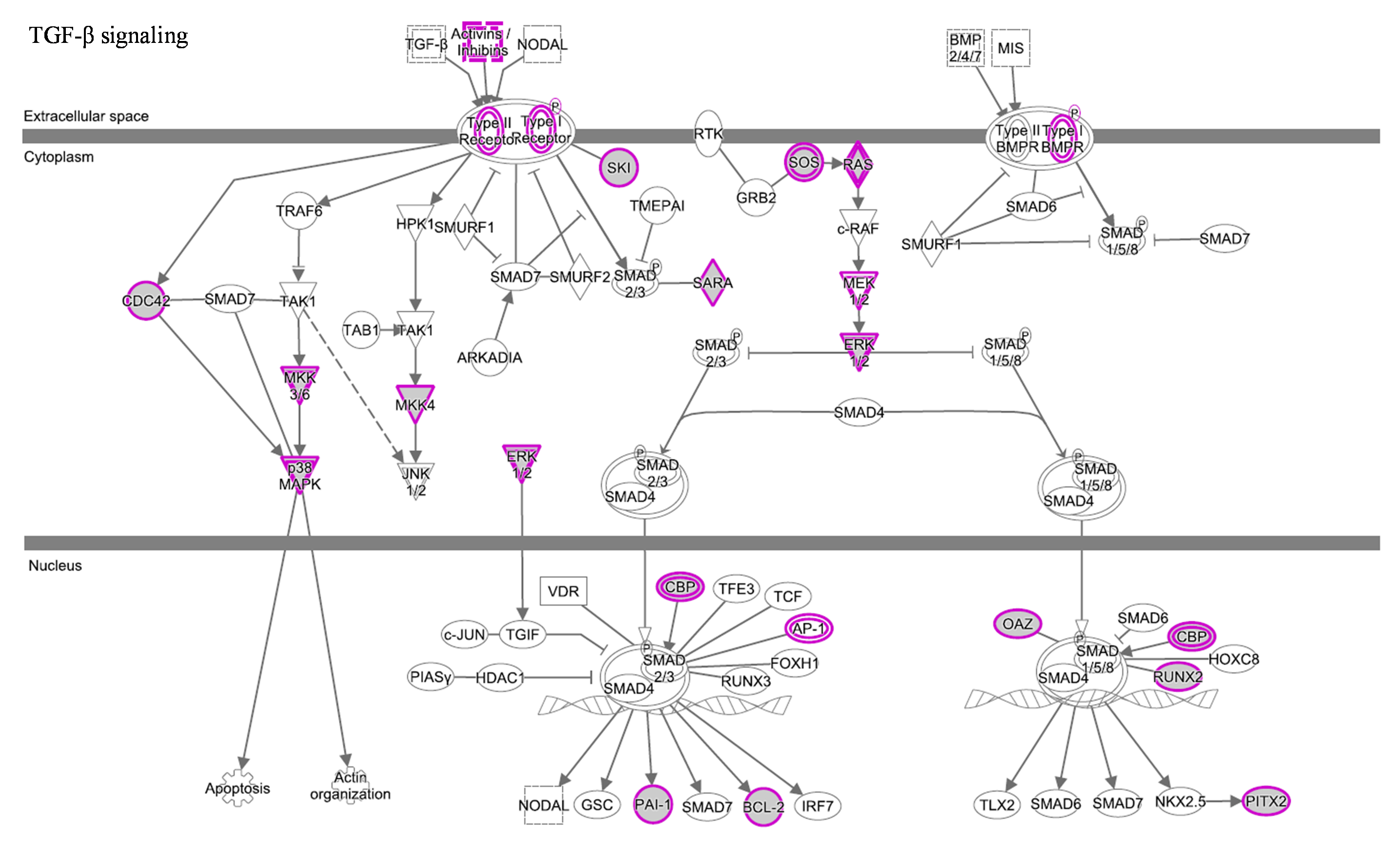


**Additional file 8.** The TGF-β signaling enriched in IPA. Objects with purple circles or triangles are acting locus by mapped genes. Abbreviations: TGF-β: Transforming Growth Factor Beta; IPA: Ingenuity Pathway Analysis.
